# Thimerosal-Induced Apoptosis in Mouse C2C12 Myoblast Cells Occurs through Suppression of the PI3K/Akt/Survivin Pathway

**DOI:** 10.1371/journal.pone.0049064

**Published:** 2012-11-07

**Authors:** Wen-Xue Li, Si-Fan Chen, Li-Ping Chen, Guang-Yu Yang, Jun-Tao Li, Hua-Zhang Liu, Wei Zhu

**Affiliations:** 1 Department of Toxicology, Guangzhou Center for Disease Control and Prevention, Guangzhou, China; 2 Faculty of Toxicology, School of Public Health, Sun Yet-sen University, Guangzhou, China; University of Pecs Medical School, Hungary

## Abstract

**Background:**

Thimerosal, a mercury-containing preservative, is one of the most widely used preservatives and found in a variety of biological products. Concerns over its possible toxicity have reemerged recently due to its use in vaccines. Thimerosal has also been reported to be markedly cytotoxic to neural tissue. However, little is known regarding thimerosal-induced toxicity in muscle tissue. Therefore, we investigated the cytotoxic effect of thimerosal and its possible mechanisms on mouse C2C12 myoblast cells.

**Methodology/Principal Findings:**

The study showed that C2C12 myoblast cells underwent inhibition of proliferation and apoptosis after exposure to thimerosal (125–500 nM) for 24, 48 and 72 h. Thimerosal caused S phase arrest and induced apoptosis as assessed by flow cytometric analysis, Hoechst staining and immunoblotting. The data revealed that thimerosal could trigger the leakage of cytochrome c from mitochondria, followed by cleavage of caspase-9 and caspase-3, and that an inhibitor of caspase could suppress thimerosal-induced apoptosis. Thimerosal inhibited the phosphorylation of Akt^ser473^ and survivin expression. Wortmannin, a PI3K inhibitor, inhibited Akt activity and decreased survivin expression, resulting in increased thimerosal-induced apoptosis in C2C12 cells, while the activation of PI3K/Akt pathway by mIGF-I (50 ng/ml) increased the expression of survivin and attenuated apoptosis. Furthermore, the inhibition of survivin expression by siRNA enhanced thimerosal-induced cell apoptosis, while overexpression of survivin prevented thimerosal-induced apoptosis. Taken together, the data show that the PI3K/Akt/survivin pathway plays an important role in the thimerosal-induced apoptosis in C2C12 cells.

**Conclusions/Significance:**

Our results suggest that in C2C12 myoblast cells, thimerosal induces S phase arrest and finally causes apoptosis via inhibition of PI3K/Akt/survivin signaling followed by activation of the mitochondrial apoptotic pathway.

## Introduction

Thimerosal is a water-soluble derivative of thiosalicylic acid. Due to its antimicrobial properties, it is widely used as a preservative in vaccines, ophthalmic products and cosmetics [1]. The safety of thimerosal has recently been questioned based on a number of studies that indicate to its possible risk of toxicity [2]–[5]. Thimerosal has been shown to cause a number of immunological and neurotoxic changes in microglia and astrocytes [1], [6]–[9], and also been shown to induce apoptosis of SK-N-SH human neuroblastoma cells via the c-Jun N-terminal kinase pathway [10] and induce DNA breaks, caspase-3 activation, membrane damage and cell death in cultured human neurons and fibroblasts [11]. Woo et al. found that it could induce G_2_/M phase arrest in human leukemia cells via the generation of reactive oxygen species and release of cytochrome C [12], while Makani et al. indicated that thimerosal could induce apoptosis in T cells via the mitochondrial pathway [13]. More recently, thimerosal has been classified as the second most common allergen after nickel [14]–[18], and also been shown to induce epithelial cytotoxicity via oxidative stress in HeLa S epithelial cells and apoptosis in human SCM1 gastric cancer cells via activation of the p38 MAP kinase and caspase-3 [19].

When people were vaccinated intramuscularly, thimerosal in vaccine directly contacts and might cause injury to skeletal muscle cells; this might be the reason for inflammation or amyotrophia at the injection site. However, little is known about the acute reactions of skeletal muscle tissues and cells following short-term exposure to thimerosal at nanomolar concentrations. Repair of degenerated muscles depends on a small group of skeletal muscle stem cells known as satellite cells [20]. Satellite cells form a group of quiescent muscle precursor cells that reside beneath the basal lamina and provide the predominant source of additional myonuclei for muscle growth [21], [22]. Once activated, satellite cells give rise to myoblasts that proliferate, differentiate, and fuse to form new muscle fibers or repair damaged muscle fibers [23], [24]. Because a pool of myoblasts available for myogenesis is important for muscle size both in vivo and in vitro [25], decreased myoblast proliferation and cytotoxicity could lead to a decrease in the number of muscle fibers. Moreover, in most cases, apoptosis serves as a physiological mechanism to remove excess myoblasts during myogenesis or muscle regeneration, while inappropriate apoptosis could pathologically lead to degeneration such as that associated with various muscular dystrophies and atrophies [26]–[29]. Because the pool of myoblasts available for myogenesis has been shown to be important for the maintenance of healthy muscle [30], [31], identification of toxins that could affect myoblasts is important in understanding the growth, disease and regeneration of skeletal muscle [29]. Several studies have found that serum starvation induces less apoptosis in differentiated myotubes than undifferentiated myoblasts and that myoblasts are more vulnerable to apoptosis than myotubes [32]. C2C12 cells, myoblastic cells that have most of the characteristics of normal myoblastic cells [33], are commonly used as a model for studying muscle cell growth and differentiation [34]–[41]. Therefore, we chose mouse C2C12 myoblast cells as a model to study the effect of thimerosal on skeletal muscles.

Apoptosis, a physiological form of cell suicide, is an important process in multicellular organisms; it ensures the elimination of superfluous tissues in development and is critical for maintenance of tissue homeostasis in adulthood [42]. The early stage of apoptosis involves death-inducing signals, including reactive oxygen and nitrogen species, expression of ligands for ‘death receptors’ and altered levels of Bcl-2 family proteins [43]. Following the early stage of apoptosis, nuclear activators, cell surface receptors, and mitochondrial pathways become activated, cells become committed to cell death, and specific cytoplasmic and nuclear events occur [44]. During this phase, caspases, which are responsible for proteolytic cleavage of a broad spectrum of cellular targets, are activated in the cytosol [43]. Apoptosis is tightly regulated by a finely tuned balance between proapoptotic and antiapoptotic factors. The Phosphoinositide 3-kinase (PI3K) pathway has been shown to play an important role in inhibition of apoptosis. Akt is an important downstream mediator of PI3K-initiated cell survival signaling. It exerts anti-apoptotic activity by preventing the release of cytochrome C from mitochondria and inactivating forkhead transcription factors. Akt also phosphorylates and inactivates the pro-apoptotic factors BAD and pro-caspase-9. PI3K/Akt plays an important role in the apoptosis of muscle cells. Factors that inhibit the PI3K/Akt pathway enhance the apoptosis of muscle cells, and when PI3K/Akt is activated, apoptosis is inhibited. Serum from scalded rats can induce apoptosis in skeletal myoblasts, and this effect can be inhibited by insulin through the PI3K/Akt signaling pathway [45]. As_2_O_3_ may exert its cytotoxicity on myoblasts by inducing apoptosis through a ROS-induced mitochondrial dysfunction, ER stress, and Akt inactivation signaling pathway [29]. In addition, dexamethasone inhibits insulin-like growth factor signaling and potentiates myoblast apoptosis [46]. However, there is less information about the relationship between thimerosal and PI3K/Akt. Recently, Akt activation has been linked to the activation of transcription of the anti-apoptotic gene survivin, a member of the inhibitor of apoptosis (IAP) gene family [47]. Survivin is the smallest member of the IAP family and has the capacity to inhibit caspase-3, caspase-7 and caspase-9. Its overexpression leads to resistance to apoptosis caused by various apoptotic stimuli [48]. Survivin is regulated by PI3K/Akt in many types of cells, and, as a member of the IAP gene family, could prevent muscle cell apoptosis [49]. There is even less information about the relationship between thimerosal and survivin.

Although it appears that mercury may induce cytotoxicity and apoptotic cell death, the underlying mechanism of this process has not been well characterized. Furthermore, there is even less information on the effect of thimerosal on muscle cells. In this study, we investigated the toxicity of nanomolar concentrations of thimerosal (125–500 nM) in mouse C2C12 myoblast cells. We found that thimerosal at nanomolar concentrations rapidly decreased cellular viability. Thimerosal induced S phase cell cycle arrest and led to apoptosis via the release of cytochrome C from mitochondria and the subsequent activation of caspase-9 and caspase-3. More importantly, we demonstrated that thimerosal treatment inactivated Akt and suppressed the expression of survivin. Blockage of the PI3K/Akt pathway by wortmannin enhanced thimerosal-induced apoptosis, and this effect could be reversed by mouse insulin-like growth factor I (mIGF-I). Furthermore, blockade of PI3K/Akt activation resulted in decreased expression of survivin, and mIGF-I could increase by activation of PI3K/Akt. The forced expression of survivin protected C2C12 cells from thimerosal-induced apoptosis, while siRNA for survivin sensitized C2C12 cells to thimerosal-induced apoptosis. Thus, inhibition of the PI3K/Akt/survivin pathway enhanced the sensitivity of C2C12 cells to thimerosal-induced apoptosis and vice versa. These data suggest that the PI3K/Akt/survivin pathway plays an essential role in the promotion of the mitochondrially mediated apoptotic process. Our study shows firstly that thimerosal induces apoptosis in mouse C2C12 myoblast cells through suppression of the PI3K/Akt/survivin pathway.

## Materials and Methods

### Reagents

Thimerosal was purchased from Sigma-Aldrich (St Louis, MO). Penicillin, streptomycin, Dulbecco's modified Eagle's medium (DMEM) and fetal bovine serum (FBS) were purchased from Gibco (Grand Island, NY). mIGF and antibodies against cytochrome C, Akt, phospho-Akt (Akt-P, Ser473), survivin, cleaved caspase-9 and cleaved caspase-3 were purchased from Cell Signaling Technology (Beverly, MA). Kits for Cycle TEST™ PLUS DNA Reagent and Annexin V-PE apoptosis were purchased from BD Biosciences (San Jose, CA). Goat anti-rabbit and anti-mouse secondary antibodies were supplied by Invitrogen (Grand Island, NY). Survivin siRNA and negative and positive controls were synthesized by Invitrogen. Wortmannin was purchased from Axxora (San Diego, CA). Z-DEVD-FMK (caspase-3 inhibitor) and Z-LEHD-FMK (caspase-9 inhibitor) were purchased from R&D Systems (Minneapolis, MN). Thimerosal (1 mM) was prepared and stored as small aliquots at −20°C for no more than 6 months and was diluted when used.

### Cell Culture

C2C12 cells were purchased from the Chinese Academy of Sciences (Shanghai, China). C2C12 cells were maintained in DMEM with 10% FBS, 100 U/mL penicillin G, and 100 µg/mL streptomycin and incubated at 37°C in a water-saturated atmosphere of 5% CO_2_.

### Establishment of stable cell lines overexpressing survivin

The retroviral vector pBabe-puro was a gift from Pro. WenChen (Faculty of Toxicology, School of Public Health, Sun Yet-sen University). The pBabe-Survivin construct was generated by RT-PCR using the sense primer GGCGGCggatccatgggagctccggcgctgcccc and the antisense primer GGCGGC gtcgacttaggcagccagctgctc. The fragment so generated was then subcloned into the retroviral vector pBabe-puro. To generate stable cell lines expressing survivin, pBabe-Survivin and pBabe-puro were introduced into C2C12 cells by retroviral infection, and transfectants were selected with puromycin (1 µg/mL).

### Transfection of cells with siRNA for survivin

C2C12 cells were plated in a 6-well plate (6×10^5^ cells/well) and transfected with survivin siRNA or control non-targeted siRNA using lipofectamine TM 2000 (Invitrogen) according to the manufacturer's instructions. Forty-eight hours after transfection, cells were collected for western blotting to confirm the effects of siRNA or incubated with thimerosal (250 nM) for an additional 24 h and then processed.

### Treatment of cells with thimerosal, wortmannin and mIGF-I

Cells were seeded in 60-mm plates at a density of 5×10^5^ cells/ml and incubated overnight. They were then treated with thimerosal (125 nM, 250 nM, and 500 nM) for 24 h, 48 h or 72 h, wortmannin (2.5 µM, 5.0 µM and 10 µM) for 24 h or mIGF-I (25 ng/mL, 50 ng/mL, and 100 ng/mL) for 24 h. For combination experiments, cells were co-treated with thimerosal (250 nM) and wortmannin (5 µM) for 24 h or with thimerosal (250 nM) and mIGF-I (10 µM) for 48 h. For thimerosal and mIGF-I, control cells were incubated with medium supplemented with sterile water; for wortmannin, control cells were incubated with DMSO at a concentration equal to that in the drug-treated cultures (final concentration 0.1%).

### Treatment of cells with thimerosal and caspase-3 and caspase-9 inhibitors

Cells were trypsinized and adjusted to the appropriate cell density (10^5^/mL). They were then plated in flasks or on 6-well plates or 3-cm dishes according to the number of cells required. After the cells had adhered to the surface of the culture plate (approximately 2 h after passage), Z-LEHD-FMK, an inhibitor of caspase-9, or the caspase-3 inhibitor Z-DEVD-FMK was added to the medium at a concentration of 50 µM. After 24 h of treatment, the cells were incubated with thimerosal at a concentration of 250 nM for an additional 48 h.

### Cell viability assay

Cell viability was determined by WST-1 assay. Briefly, cells were seeded at a density of 5×10^3^ cells/100 µL in 96-well plates and cultured overnight. The medium was then exchanged for fresh medium containing 125 nM, 250 nM or 500 nM thimerosal for 24, 48, or 72 h. After the treatment, 10 µL WST-1 solution (Roche Diagnostics, Laval, Quebec, Canada) was added to each well, and the cells were incubated for an additional 2 h at 37°C. The absorbance was determined using a microplate reader at a test wavelength of 450 nm and a reference wavelength of 630 nm.

### Cell cycle arrest assay

The percentage of cells in various phases of the cell cycle was assayed according to the instruction manual of Flow Cytometry. At the indicated time points, cells were trypsinized and fixed in 70% ethanol followed by staining with PI buffer (20 µg/mL PI, 0.1% Triton X-100 and 200 µg/mL RNase A in PBS). The stained cells were analyzed using a FACSCalibur flow cytometer (BD Biosciences); subsequent data analysis was conducted using Modfit LT software.

### Apoptosis assay

For Hoechst 33258 staining, cells were grown in 24-well plates and treated as described above. Nuclear DNA in treated cells was visualized by staining with the DNA-specific dye Hoechst 33258 at a final concentration of 6 µg/mL. The cells were observed immediately with filters for blue fluorescence.

For flow cytometric analysis, cells were exposed to thimerosal at the indicated concentrations for 24 or 48 h. The cells were then harvested, washed twice with cold PBS, and resuspended in binding buffer at 1×10^5^ cells/mL. A 100-µl aliquot of the suspension was incubated with 5 µl of Annexin V-PE and 5 µl of propidium iodide (20 µg/mL) in the dark for 20 min at room temperature. Finally, 400 µL of binding buffer was added to each sample, and the samples were analyzed by flow cytometry within one hour.

### Western blot analysis

For immunoblotting, cells were pelleted by centrifugation at 320×g for 10 min and resuspended in lysis buffer (20 mM Tris-HCl pH 7.4, 2 mM EDTA, 500 µM sodium orthovanadate, 1% Triton X-100, 0.1% SDS, 10 mM NaF, 10 µg/mL leupeptin, and 1 mM PMSF). Aliquots (20 µg) of the lysates were separated on a 12% SDS-polyacrylamide gel and transferred electrophoretically (Bio-Rad) to a PVDF membrane (Millipore, USA). Blots were blocked for 2 h in blocking buffer (5% non-fat dry milk in TBST buffer) and incubated with primary antibodies (1∶1000) overnight at 4°C. After washing with PBST (10 mM phosphate buffer, 2.7 mM KCl, 140 mM NaCl and 0.05% Tween 20, pH 7.4), the appropriate HRP-conjugated secondary antibody (1∶5000) was added to the preparation. The blot was incubated at 37°C for 1 h and developed using an enhanced chemiluminescence detection system (Beyotime Institute of Biotechnology, China).

### Statistical analysis

The data are presented as the mean ± standard deviation. SPSS 11.0 software was used for statistical analysis. The data for time and dosage effects were analyzed using two-way ANOVA. When appropriate, data were analyzed using one-way analysis of variance (ANOVA). The a priori significance level was set at P<0.05.

## Results

### Thimerosal significantly inhibited the proliferation of mouse C2C12 myoblast cells

**Figure 1:**
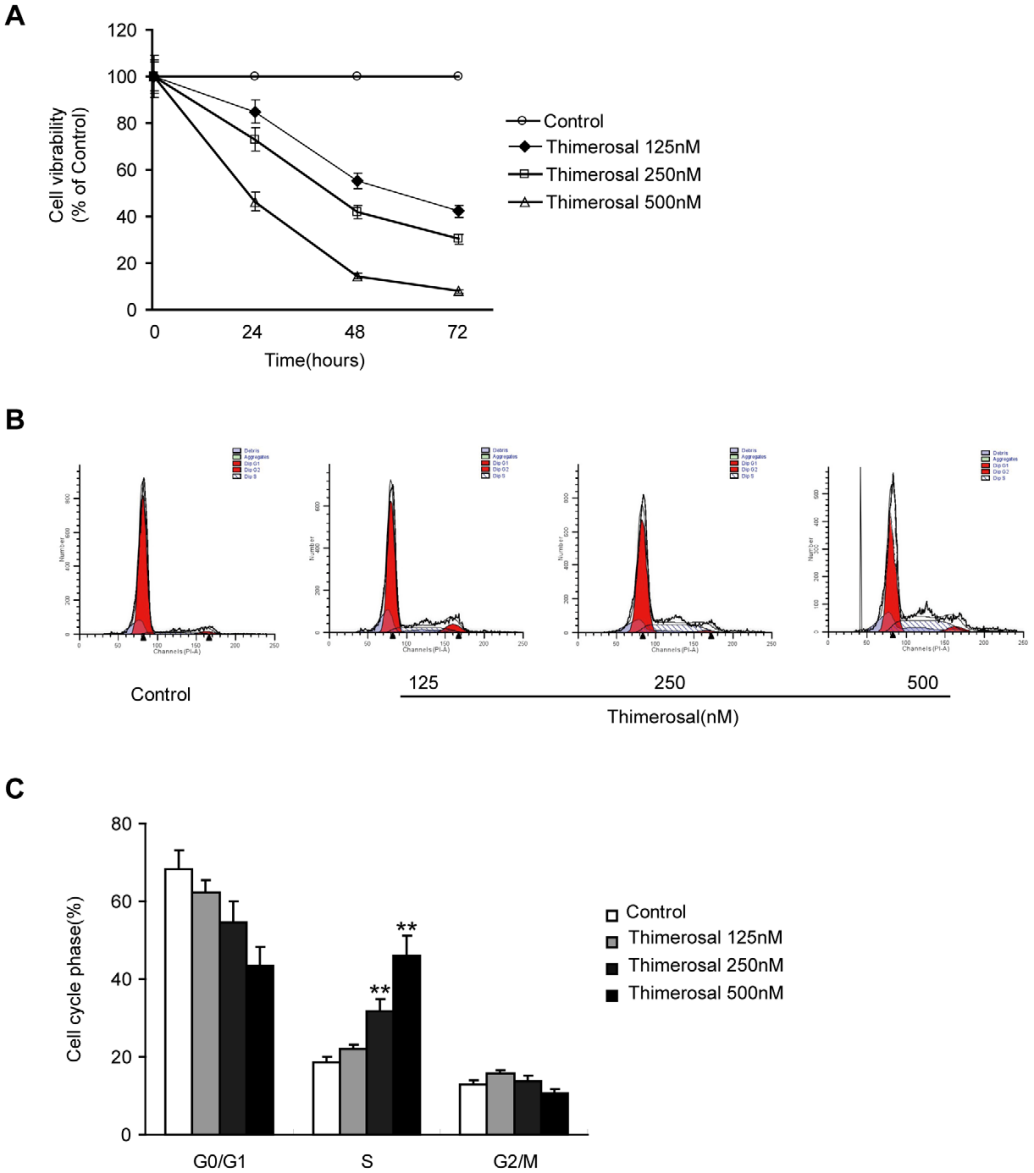
Effects of thimerosal on proliferation of C2C12 myoblast cells. C2C12 myoblast cells were treated with thimerosal (125, 250 and 500 nM) for 24, 48 or 72 h. **A.** C2C12 myoblast cell viability determined by WST-1. **B.** Cell cycle distribution of C2C12 myoblast cells analyzed by flow cytometry. **C.** The percentage of cells in the various phases of the cell cycle. All data are reported as the mean±S.E.M. of three independent experiments. **P<0.01 compared with control.

The effects of thimerosal on the proliferation of C2C12 cells are shown in Figures 1A. Thimerosal treatment (125, 250 and 500 nM for 24, 48 and 72 h) resulted in time- and dose-dependent inhibition of cell proliferation. Treatment of cells with 500 nM thimerosal for 24 h reduced cell proliferation by more than 50%, while treatment with 500 nM thimerosal for 48 h completely abolished C2C12 cell proliferation. This inhibition of cell proliferation could be due to cell cycle arrest and induced apoptotic cell death.

We found that exposure to thimerosal resulted in accumulation of cells in the S phase of the cell cycle. Cell cycle analysis performed after 48-hour incubation of cells in increasing concentrations of thimerosal (125, 250 and 500 nM) showed that this agent arrested the cells in S phase in a dose-dependent manner (Figures 1B and 1C). After exposure to 500 nM thimerosal, 50.6% of the cells were in S phase, whereas only 10.9% of the cells were in G2/M phase and 38.5% were in G0/G1 phase. In contrast, in the control cell cycle distribution, the S-phase fraction was 20.5% (Figures 1B and 1C).

### Thimerosal induced apoptosis in mouse C2C12 cells

**Figure 2:**
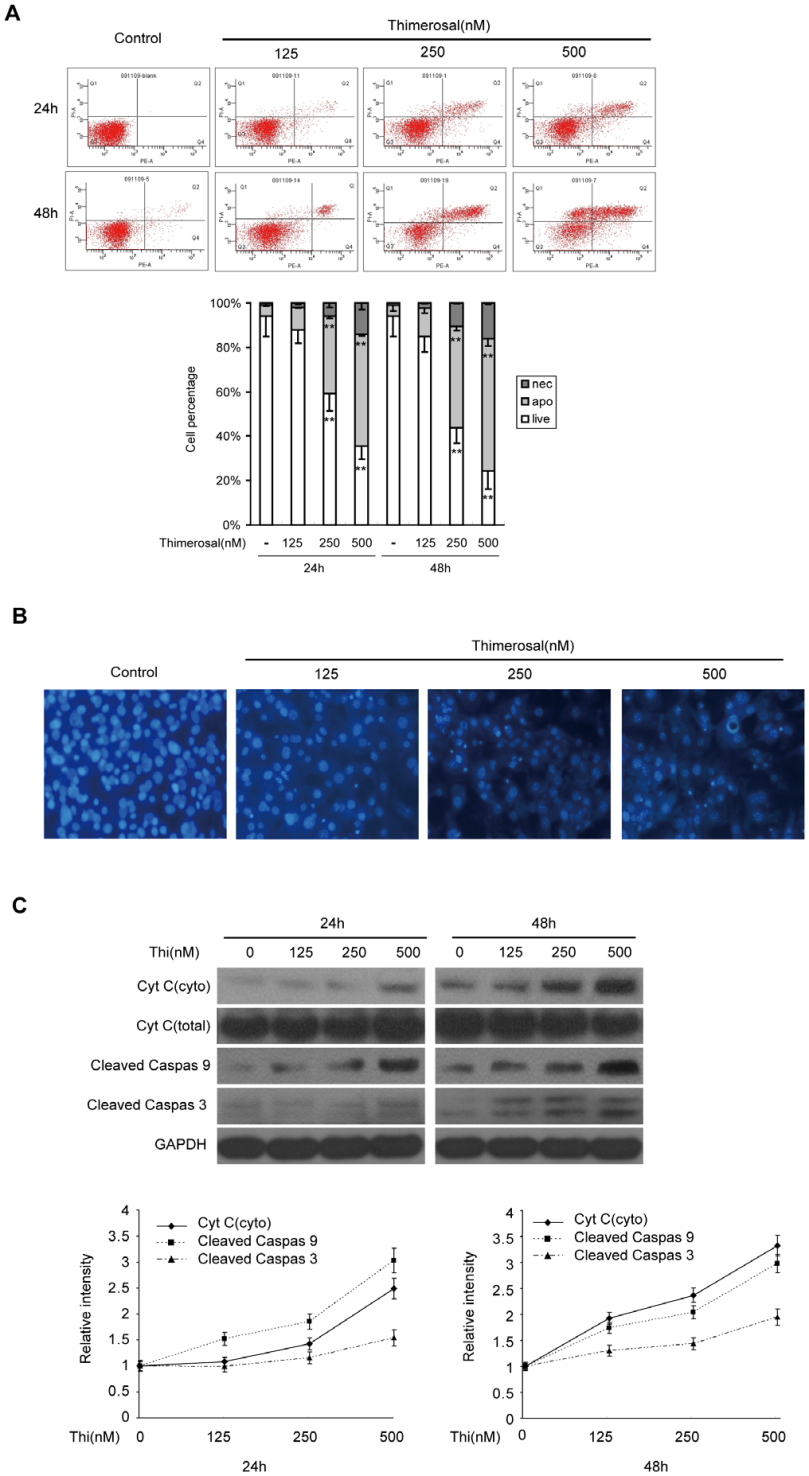
Thimerosal induced apoptosis in C2C12 myoblast cells. C2C12 myoblast cells were treated with thimerosal (125, 250 and 500 nM) for 24 or 48 h. **A.** After treatment, cells were stained with Annexin V-FITC/propidium iodide and measured by flow cytometry. The columns illustrate the flow cytometric results. **B.** After exposure to thimerosal for 48 h, cells were stained with Hoechst 33258 and observed under a fluorescence microscope. The magnification was ×200. **C.** After treatment, proteins from total cell lysates were separated by SDS-PAGE gel electrophoresis and immunoblotted with antibodies against cytochrome c, cleaved caspase-9, cleaved caspase-3 and GAPDH followed by densitometric quantification. Data are means±S.E.M. of values from three independent experiments. **P<0.01 vs. control.

To investigate thimerosal-induced apoptosis, C2C12 cells were treated with thimerosal for varying periods of time and apoptosis was evaluated by flow cytometric analysis and Hoechst staining. The results of cell flow cytometry are shown in Figures 2A. Thimerosal at concentrations of 125, 250 and 500 nM for 24 and 48 h produced significantly more apoptosis (250 nM, 24 h, 34.9±0.2%; 250 nM, 48 h, 45.8±0.3%) in a time- and dose-dependent manner than occurred in untreated control C2C12 cells (P<0.05 or P<0.01).

Apoptotic cells show nuclear condensation and DNA fragmentation, which can be detected by Hoechst staining and fluorescence microscopy. In separate experiments, bisbenzimide (Hoechst 33258) staining of chromatin was used to distinguish and quantify apoptotic cells. The cells were treated with thimerosal at concentrations of 125 nM, 250 nM and 500 nM for 48 h and then stained with Hoechst 33258. As shown in Figures 2B, thimerosal-treated cells exhibited typical apoptotic morphology with condensation and fragmentation of the nucleus. These experiments provide strong evidence that apoptosis is induced in mouse C2C12 cells after thimerosal treatment.

The mitochondrial pathway is a very important pathway in thimerosal-induced apoptosis [13]. To explore the mechanism by which thimerosal induces apoptosis in C2C12 cells, we measured the release of cytochrome C from mitochondria and the cleavage of caspase-3 and caspase-9. Figures 2C and 2D showed that there was increased release of cytochrome C from the mitochondria of thimerosal-treated cells. The percentage of cytochrome C released at 24 h increased 1.1 (125 nM), 1.4 (250 nM) and 2.5 (500 nM) times over that of control cells; at 48 h, it increased 1.9 (125 nM), 2.3 (250 nM) and 3.4 (500 nM) times. Release of cytochrome C into the cytosol leads to the initiation of the caspase cascade, primarily through the activation of caspase-9 and caspase-3 [27], [43]. Figures 2C and 2D show that thimerosal activated both caspase-9 and caspase-3 in a concentration-dependent manner. These results suggest that thimerosal activates caspase via the mitochondrial pathway.

### Thimerosal inhibited the levels of phosphorylated Akt^ser473^, and wortmannin could sensitize cells to thimerosal-induced apoptosis

**Figure 3:**
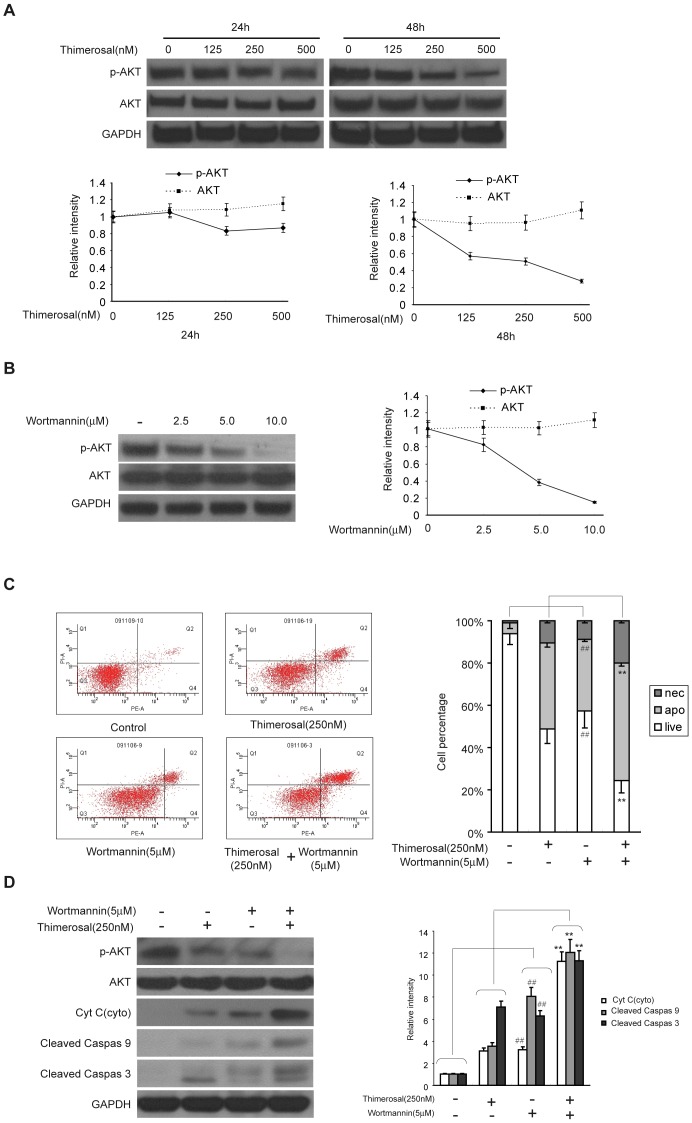
Wortmannin, a specific inhibitor of PI3K, enhanced the sensitization of C2C12 myoblast cells to thimerosal-induced apoptosis. **A.** C2C12 myoblast cells were treated with thimerosal (125 nM, 250 nM and 500 nM) for 24 or 48 h. Cells were lysed, and the expression of Akt and pAkt^Ser473^ were assayed by western blot analysis. GAPDH was used as a loading control, and the blots were quantified by densitometry. **B.** C2C12 myoblast cells were treated with wortmannin at concentrations of 2.5, 5.0 or 10 µM for 24 h. Expression of total Akt and pAkt^Ser473^ was assayed by western blot analysis and densitometry. **C.** Cells were co-treated with thimerosal (250 nM) and wortmannin (5 µM) for 24 h and stained with annexin V-FITC/propidium iodide. The columns illustrate the flow cytometric results. **D.** After co-treatment, proteins from total cell lysates were separated by SDS-PAGE gel electrophoresis and immunoblotted with antibodies against Akt, pAkt^Ser473^, cytochrome c, cleaved caspase-9, cleaved caspase-3 and GAPDH followed by densitometric quantification. Data are means±S.E.M. of values from three independent experiments. **P<0.01 vs. single treatment with 250 nM thimerosal; ## P<0.01 vs. untreated cells.

The PI3K/Akt pathway mediates the effects of extracellular signals on various cellular processes including growth, differentiation, survival, and metabolism [1]–[3]. It has been reported that exposure of cells to some types of heavy metals can lead to apoptosis through inactivation of an Akt-related pathway [9]. In our study, as shown in Figures 3A, thimerosal effectively decreased the phosphorylation of AKT in myoblasts in a dose-dependent manner. The percentage of phosphorylated Akt^ser473^ decreased to 56.2%±2.5% (125 nM), 51.3%±3.2% (250 nM) and 27.4%±2.2% (500 nM) compared with control cells after 48 h treatment. As shown in Figures 3B, wortmannin, an inhibitor of PI3K, significantly decreased pAkt^ser473^ in C2C12 cells to 81.2%±5.4% (2.5 µM), 36.7%±3.2% (5 µM) and 13.1%±1.5% (10 µM) compared with control cells after 48 h treatment. The effect of wortmannin on thimerosal-induced apoptosis was evaluated by flow cytometric analysis, and the release of cytochrome C and the activation of caspase-9 and caspase-3 were detected by western blot. As shown in Figures 3C, combination treatment significantly increased the percentage of apoptotic cells (55.6%, 5 µM wortmannin and 250 nM thimerosal for 48 h) compared with wortmannin (33.9%±2.5%) and thimerosal (40.0%±3.3%) alone. As shown in Figures 3D, similar results were obtained by western blot. The level of cytochrome C in the cytoplasm of cells undergoing combined treatment was 11.1 times more than that in the cytoplasm of control cells, compared with wortmannin (4.5 times) and thimerosal (4.1 times) alone. Thus, the combination treatment clearly activated caspase-3 and caspase-9 and was more effective than either wortmannin or thimerosal alone.

### Activation of the PI3K signaling pathway protected C2C12 cells from apoptosis induced by thimerosal

**Figure 4:**
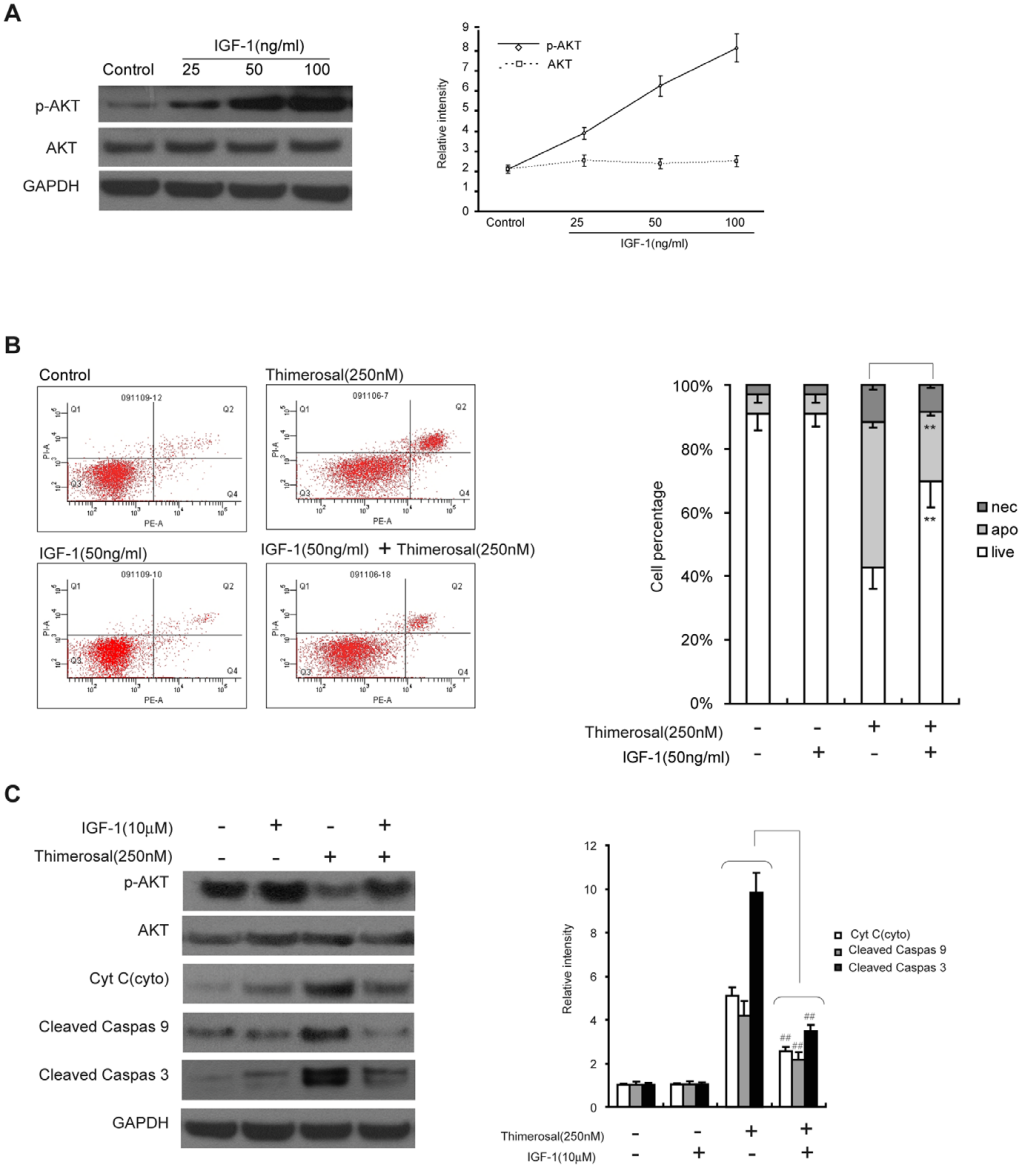
Activation of PI3K/Akt signaling inhibited thimerosal-induced apoptosis in C2C12 myoblast cells. **A.** C2C12 cells were treated with mIGF-I at concentrations of 25, 50, or 100 ng/mL for 24 h. Expression of total Akt and pAkt^Ser473^ was assayed by western blot analysis followed by densitometry. **B.** Cells were co-treated with thimerosal (250 nM) and mIGF-I (50 ng/mL) for 48 h and stained with Annexin V-FITC/propidium iodide followed by flow cytometry. **C.** After co-treatment, proteins from total cell lysates were separated by SDS-PAGE gel electrophoresis and immunoblotted with antibodies against Akt, pAkt^Ser473^, cytochrome c, cleaved caspase-9, cleaved caspase-3 and GAPDH followed by densitometric quantification. Data are means±S.E.M of values from three independent experiments. **P<0.01 vs. single treatment with 250 nM thimerosal.

Because an inhibitor of PI3K enhanced the sensitization of C2C12 cells to thimerosal-induced apoptosis, we asked whether activation of the PI3K signaling pathway could protect C2C12 cells from apoptosis induced by thimerosal. C2C12 cells were first exposed to different concentrations of mIGF-I (25, 50 and 100 ng/ml) for 48 h. As shown in Figures 4A, mIGF-I significantly increased the level of pAkt^ser473^; exposure to mIGF-I (50 ug/mL) increased the level of pAkt^ser473^ to 5.5-fold that of control cells. The cells were then treated with thimerosal (250 nM) and/or mIGF-I (50 ng/mL) for 48 h. As shown in Figures 4B, activation of the PI3K/Akt pathway by mIGF-I (21.8%±2.2%, mIGF-I 50 µg/ml) decreased the apoptosis induced by thimerosal (48.8%±3.2%, thimerosal 250 nM); As shown in Figures 4C, similar results were obtained by western blot; mIGF-I (50 ug/mL) decreased cytochrome C release induced by thimerosal (250 nM) from 5.3 times to 2.1 times that of control cells. As observed from the figure, combination treatment also reduced the activation of caspase-3 and caspase-9 by thimerosal.

### Thimerosal decreased the expression of survivin via the PI3K/Akt pathway

**Figure 5:**
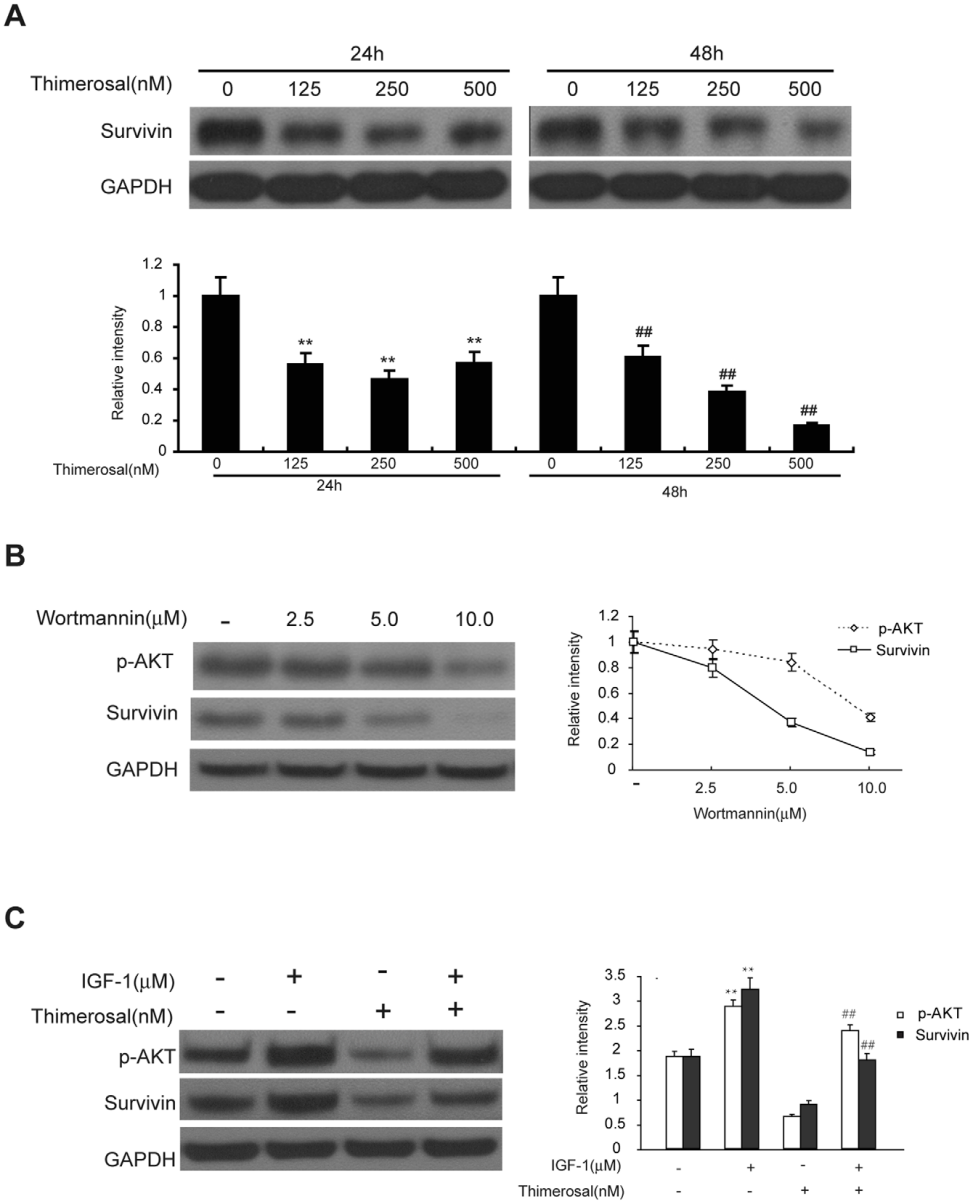
Thimerosal decreases the expression of survivin via the PI3K/Akt pathway. **A.** C2C12 myoblast cells were treated with thimerosal (125 nM, 250 nM or 500 nM) for 24 or 48 h. Cells were lysed, and the expression of survivin was assayed by western blot analysis and densitometry. GAPDH was used as a loading control. **B.** C2C12 cells were treated with wortmannin at concentrations of 2.5, 5.0 and 10 µM for 24 h. The expression of survivin was assayed by western blot analysis followed by densitometry. **C.** Cells were co-treated with thimerosal (250 nM) and mIGF-I (50 ng/mL) for 48 h, and survivin expression was quantitated by western blotting and densitometry. Data are means±S.E.M of the values from three independent experiments.

Survivin, the smallest member of the IAP family, has the capacity to inhibit caspase-3, caspase-7, and caspase-9, and its overexpression confers resistance to apoptosis caused by various apoptotic stimuli [48], [50]. Given the importance of the PI3K/Akt/survivin pathway in apoptosis and cell survival, we next determined whether thimerosal could inhibit the expression of survivin through inactivation of the PI3K/Akt/survivin pathway. As shown in Figures 5A, exposure of C2C12 cells to thimerosal at concentrations of 125, 250 and 500 nM for 24 and 48 h significantly decreased the level of survivin protein in a time- and dose-dependent manner compared to untreated control C2C12 cells (P<0.05 or P<0.01). Wortmannin (2.5, 5.0, 10 µM for 24 h), an inhibitor of PI3K, inhibited the expression of survivin in C2C12 cells (Figures 5B), and mIGF-I (50 ng/mL for 24 h) increased the level of survivin protein and protected cells from the inhibitory effect of thimerosal on survivin expression (Figures 5C).

### Survivin siRNA sensitizes cells to thimerosal-induced apoptosis

**Figure 6:**
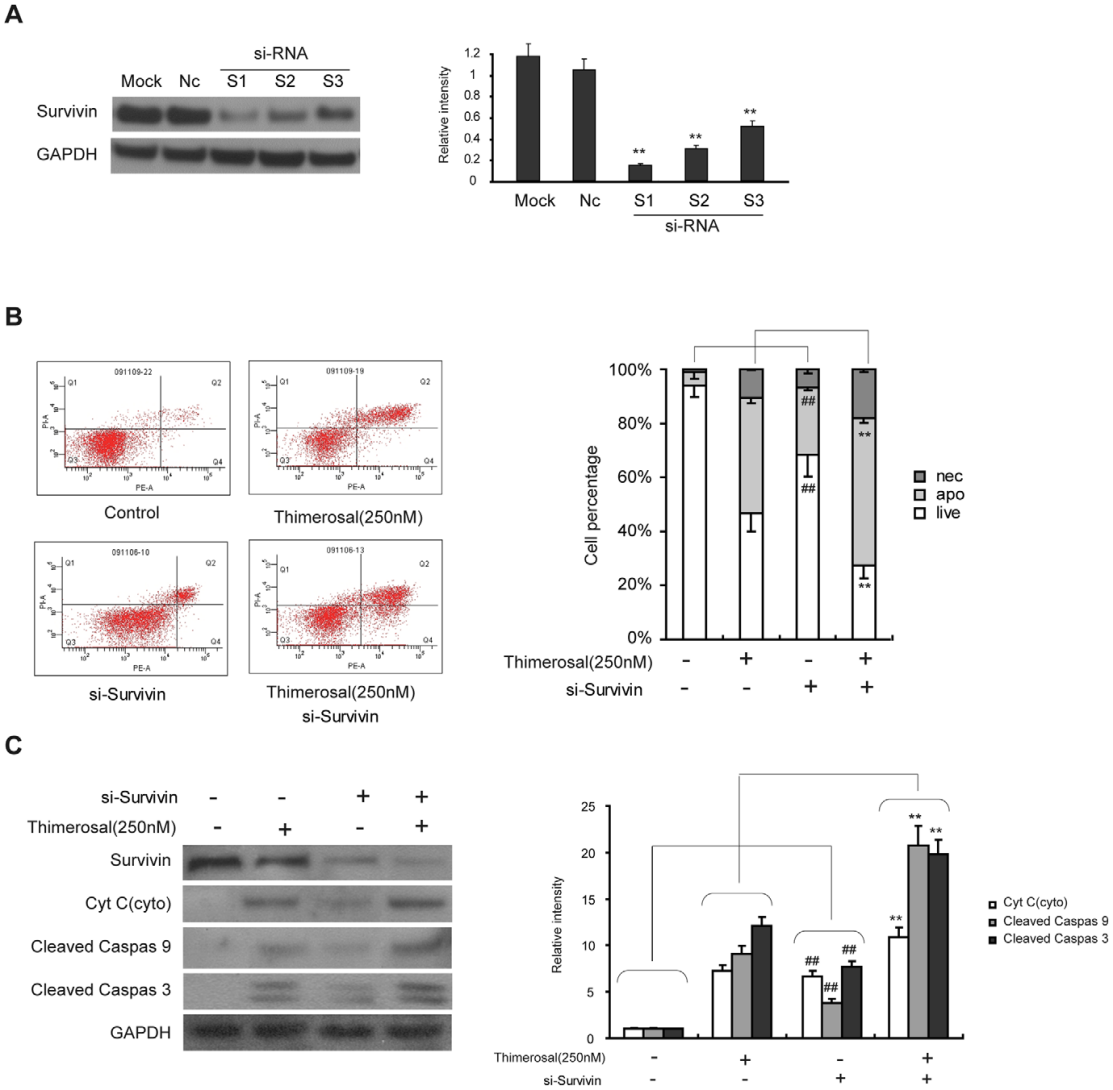
SiRNA against survivin enhanced the sensitization of C2C12 myoblast cells to thimerosal-induced apoptosis. **A.** After 48 h transfection with non-targeted (NC) or survivin (S1, S2 and S3) siRNA or mock transfection without siRNA (MOCK), the expression of survivin was assayed by western blot analysis and densitometry. **P<0.01 vs. non-targeted (NC) **B.** After transfection with non-targeted (NC) or survivin S1 siRNA for 48 h, cells were treated with or without thimerosal (250 nM) for 24 h and stained with Annexin V-FITC/propidium iodide followed by flow cytometry. **C.** After treatment of siRNA and thimerosal, proteins from total cell lysates were separated by SDS-PAGE gel electrophoresis and immunoblotted with antibodies against survivin, cytochrome c, cleaved caspase-9, cleaved caspase-3 and GAPDH followed by densitometric quantification. Data are means±S.E.M of the values from three independent experiments. **P<0.01 vs. single treatment with 250 nM thimerosal; ## P<0.01 vs. untreated cells.

The results described in the previous section show that exposure of cells to thimerosal can decrease the expression of survivin through the PI3K/Akt pathway. To better understand the effects of survivin on thimerosal-induced apoptosis, we used survivin siRNA. Three different siRNA duplexes were tested; S1 had the highest efficiency (Figures 6A) and was used in the experiments. As shown in Figures 6B, depletion of survivin in C2C12 cells by S1 siRNA significantly increased the apoptosis rate (54.6%±1.9%, survivin siRNA and thimerosal (250 nM for 48 h)) compared with survivin siRNA (24.9%±1.5%) and thimerosal (42.8%±2.6%) alone. Similar results, shown in Figures 6C, were obtained by western blot. With combined treatment, the level of cytochrome C in cytoplasm was 12.1-fold that of control cells; with survivin-siRNA alone it was 5.2-fold, and with thimerosal alone it was 5.8-fold that of control cells. Thus, combination treatment activated caspase-3 and caspase-9 more effectively than either survivin siRNA or thimerosal alone.

### Survivin overexpression protects C2C12 cells from apoptosis induced by thimerosal

**Figure 7:**
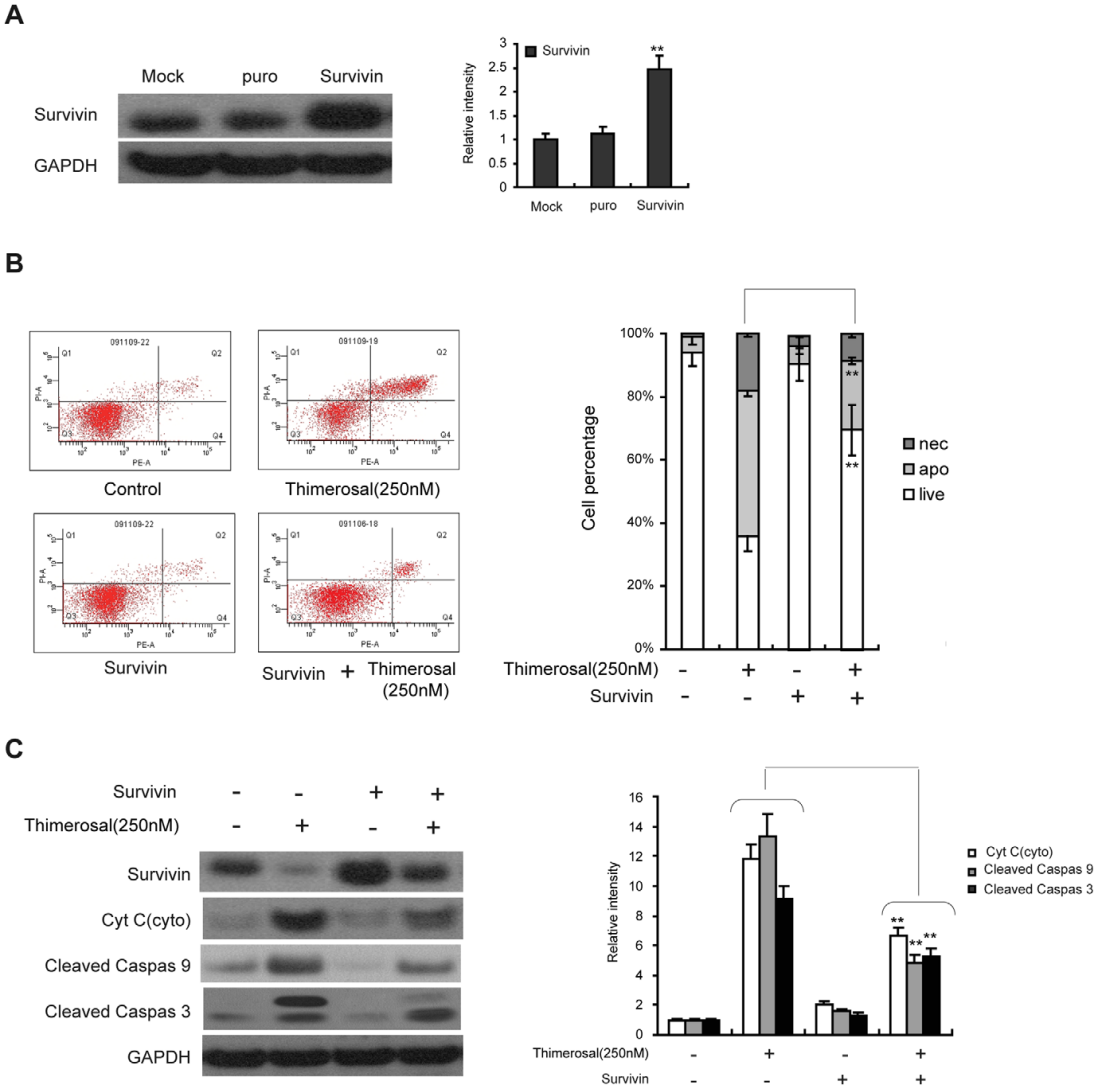
Overexpression of survivin inhibited thimerosal-induced apoptosis in C2C12 myoblast cells. **A.** C2C12-survivin with overexpression of survivin, C2C12-puro with control vector and normal C2C12 cells (Mock) were subjected to western blot analysis with antibody against survivin followed by densitometric quantification. **P<0.01 vs. C2C12-puro **B.** C2C12-survivin and C2C12-puro were treated with or without thimerosal (250 nM) for 48 h and stained with Annexin V-FITC/propidium iodide followed by flow cytometry. **C.** After treatment with or without thimerosal, proteins from C2C12-survivin and C2C12-puro were analyzed by immunoblotting with antibodies against survivin, cytochrome c, cleaved caspase-9, cleaved caspase-3 and GAPDH followed by densitometric quantification. Data are means±S.E.M of the values from three independent experiments. **P<0.01 vs. single treatment with 250 nM thimerosal.

Because depletion of survivin sensitized C2C12 cells to thimerosal-induced apoptosis, we asked whether overexpression of survivin could protect C2C12 cells from apoptosis induced by thimerosal. A cell line with forced expression of survivin, C2C12-survivin, was established using a retroviral vector, and the expression of survivin was detected by western blot. As shown in Figures 7A, C2C12-survivin cells exhibited a higher level of survivin expression than mock-infected cells or puro control cells. C2C12-survivin cells were treated with thimerosal (250 nM) for 48 h. As shown in Figures 7B, overexpression of survivin significantly decreased apoptosis induced by thimerosal from 46.8%±1.2% to 22.8%±2.3%. Similar results were obtained by western blotting (Figures 7C). Overexpression of survivin decreased the amount of cytochrome C released into the cytosol after exposure to thimerosal (250 nM) from 11.3-fold to 6.1-fold that of control cells. C2C12-survivin cells also showed a lower level of cleavage activation of caspase-3 and caspase-9 after thimerosal exposure than control cells.

### Inhibitors of caspase-9 (Z-LEHD-FMK) or caspase-3 (Z-DEVD-FMK) reduced thimerosal-induced apoptosis in C2C12 cells

**Figure 8:**
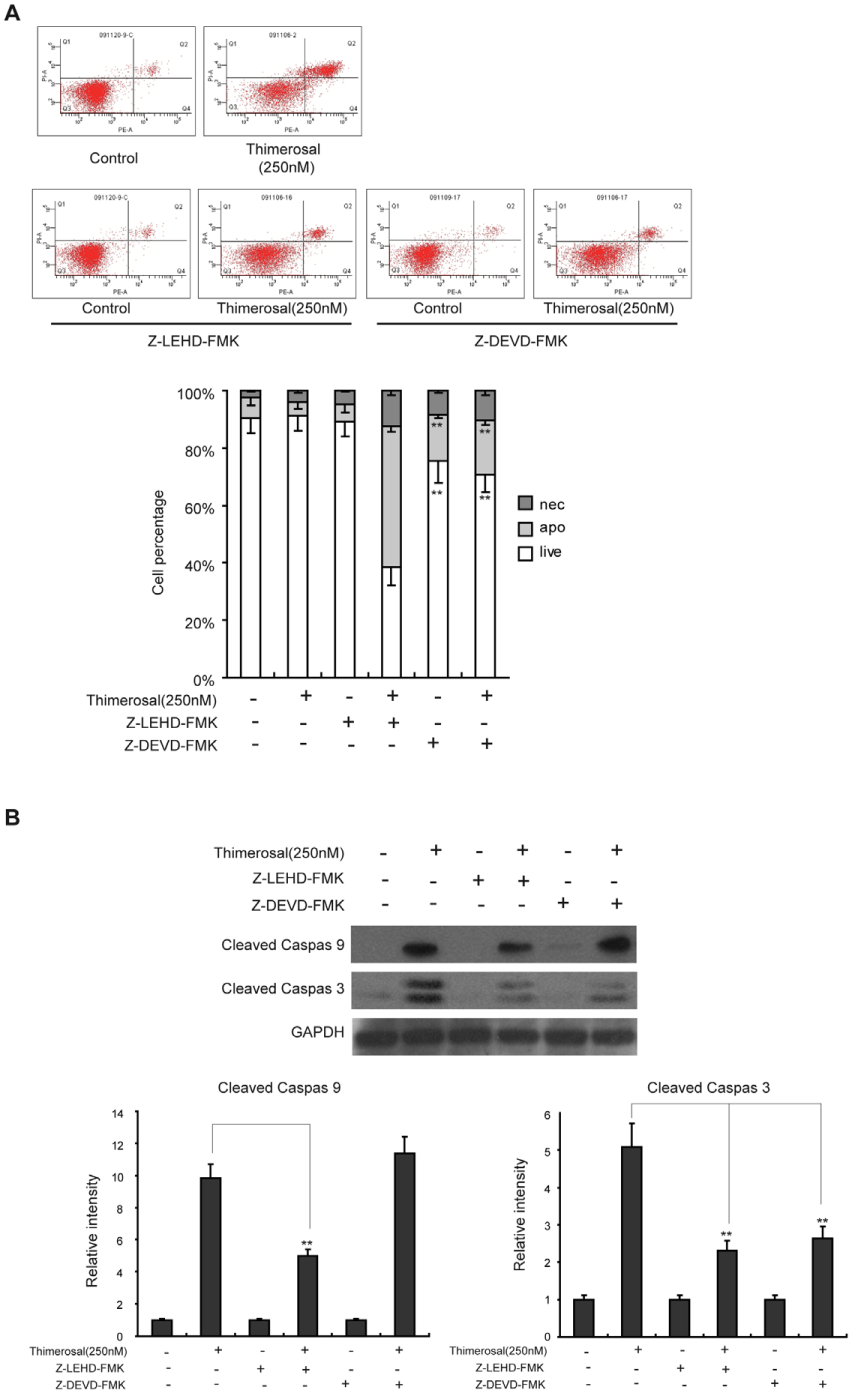
The effect of Z-LEHD-FMK and Z-DEVD-FMK on thimerosal-induced apoptosis in C2C12 myoblast cells. C2C12 cells were treated with Z-LEHD-FMK, an inhibitor of caspase-9, or with the caspase-3 inhibitor Z-DEVD-FMK at a concentration of 50 µM for 24 h and then incubated with or without thimerosal (250 nM) for 48 h. **A.** After treatment, cells were stained with Annexin V-FITC/propidium iodide followed by flow cytometry. **B.** After treatment, proteins from total cell lysates were separated by SDS-PAGE gel electrophoresis and immunoblotted with antibodies against cleaved caspase-9, cleaved caspase-3 and GAPDH followed by densitometric quantification. Data are means±S.E.M of the values from three independent experiments. **P<0.01 vs. single treatment with 250 nM thimerosal.

To confirm the role of caspase activation in thimerosal-induced apoptosis, the caspase inhibitors ZLEHD-fmk (a caspase-9 inhibitor) and ZDEVD-fmk (a caspase-3 inhibitor) were used. Cells were treated with ZLEHD-fmk (10 µM) or ZDEVD-fmk (10 µM) for 24 h and then with thimerosal (250 nM) for 48 h. Figures 8A shows that ZLEHD-fmk and ZDEVD-fmk prevented cell apoptosis induced by thimerosal. ZLEHD-fmk decreased apoptosis induced by thimerosal from 48.8%±1.2% to 18.8%±2.8%, while ZDEVD-fmk decreased apoptosis from 48.8%±3.2% to 15.6%±2.7%. As shown in Figures 8B, ZLEHD-fmk inhibited the cleavage activation of caspase-9 and caspase-3, and ZDEVD-fmk inhibited the cleavage activation of caspase-3. These results indicate that cleavage activation of caspase-9 and caspase-3 might play an important role in apoptosis induced by thimerosal in C2C12 cells.

## Discussion

Thimerosal is widely used as a preservative. In vaccine products, it is typically used at a concentration of 0.0002–0.01%, which is equal to 5–250 µM in molarity [51]. Thimerosal is also used in cosmetics, often at a concentration of 0.0007%, equal to 17.5 µM in molarity [52]. In our study, we used thimerosal at final concentrations of 125–500 nM, lower than the concentrations of thimerosal typically used in cosmetics and vaccines. Therefore, our results provide meaningful information on the possible effects of the use of thimerosal in practical applications. Our study showed that short-term exposure to thimerosal at low concentrations could induce apoptosis in myoblast cells and suggest that exposure to thimerosal may be an important risk factor for skeletal muscle cell development and growth.

Mercury vapor and methylmercury (MeHg) are both neurotoxic [53] and are known to have adverse effects on the developing brain during exposure in utero [54]. However, the toxicology of thimerosal is less well understood than that of MeHg. Although thimerosal has been shown to induce apoptosis in some cell types, until now, there has been very little information about the effect of thimerosal on muscle cells. Our study showed that thimerosal causes cell cycle arrest and apoptosis of mouse C2C12 myoblast cells through inhibition of the PI3K/Akt/survivin pathway and the activation of the mitochondrial apoptotic pathway, including the release of cytochrome C followed by activation of caspase-9 and caspase-3.

Myoblasts dictate skeletal muscle size, and adequate numbers of myoblasts are required for precise muscle regeneration [55], [56]. Because myoblasts play an essential role in muscle tissue, mouse C2C12 myoblast cells were used to study the effects of thimerosal on muscle cells in vitro. As mentioned, thimerosal has been shown to promote apoptosis in some cell types. In the present study, we found that treatment with thimerosal (125–500 nM for 24–72 h) significantly decreased cell viability and increased cell apoptosis in a dose-dependent manner in mouse C2C12 myoblast cells. Woo et al. found that thimerosal induced G2/M phase arrest in human leukemia cells [12], and we found that thimerosal induces S phase arrest in C2C12 cells. This suggests that thimerosal may cause cell cycle arrest by different molecular mechanisms in different cell lines; more research is needed to resolve this question.

Mitochondria play a crucial role in regulating apoptotic cell death [57]. When a high ratio of Bax to Bcl-2 causes a loss of mitochondrial membrane potential, cytochrome C is released from the intermembrane space of mitochondria to the cytosol, where it triggers the formation of apoptosome-containing apoptotic protease and activates caspase-9. Caspase-9 activates procaspases, including procaspase-3, which carries out the process of apoptosis [58], [59]. Humphrey et al. found that mitochondria mediated thimerosal-induced apoptosis in a human neuroblastoma cell line (SK-N-SH) [7], and Makani et al. found that the mitochondrial pathway formed the biochemical and molecular basis of thimerosal-induced apoptosis in T cells [13]. In our study, the mitochondrially mediated apoptosis pathway was activated when mouse C2C12 myoblast cells were treated with thimerosal. The treatment triggered cytochrome C release into the cytoplasm, which in turn activated caspase-9 and subsequently caspase-3, leading to an increase in apoptosis. A specific inhibitor of caspase-9, Z-LEHD-FMK, decreased the expression of cleaved caspase-9 and cleaved caspase-3, while a specific inhibitor of caspase-3, Z-DEVD-FMK, decreased the expression of cleaved caspase-3 but not cleaved caspase-9. These results suggest that a mitochondria-dependent pathway is involved in thimerosal-induced apoptosis in C2C12 myoblasts.

The PI3K/Akt signaling pathway plays a central role in diverse cellular functions, including proliferation, apoptosis, cell survival and metabolism. The PI3K/Akt pathway has been reported to play a critical role in the inhibition of apoptosis [60], [61]. Overexpression of Akt has been found to prevent apoptosis in many cell types and to result in a resistance to or delay of cell death [62]. Akt may regulate apoptosis by phosphorylation and inactivation of the Bcl-2 family member BAD, which controls the release of cytochrome C from mitochondria [63]. Akt could also inhibit cell apoptosis by inactivation of caspase-9 and FKHRL1, a member of the Forkhead family of transcription factors [64]. There is very little published research on the effects of thimerosal on the PI3K/Akt pathway. In the work presented in this paper, we found that thimerosal significantly decreased the level of pAkt^Ser473^ in a dose- and time-dependent manner without changes in total Akt protein. Inhibiting Akt activation with wortmannin, which blocks activation of PI3K, depressed the expression of pAkt^Ser473^ and sensitized thimerosal-induced apoptosis. We also found that overactivation of PI3K/Akt with mIGF-I suppressed thimerosal-induced apoptosis. Our results suggest that PI3K/Akt may be involved in thimerosal-induced cell apoptosis in C2C12 cells and that Akt may play a protective (anti-apoptotic) role in thimerosal-induced C2C12 myoblasts. Thimerosal may inhibit the activation of PI3K/Akt by reactive oxygen species (ROS). Thimerosal could induce oxidative stress in some types of cells. Lee et al. found that thimerosal could induce oxidative stress in Hela S epithelial cells and that ROS play a pivotal role in thimerosal-mediated cell death in these cells [51]. Makani et al. suggested that thimerosal induced apoptosis in T cells via the mitochondrial pathway by inducing oxidative stress and depletion of GSH [13]. ROS could inhibit the phosphorylation of Akt and trigger cytotoxicity; H_2_O_2_ induction of ROS has been shown to inhibit the phosphorylation of Akt [65]. Yuan et al. found that arsenic induced apoptosis in C2C12 myoblasts through a reactive oxygen species-induced mitochondrial dysfunction and inactivation of the Akt signaling pathway [29]. The mechanism through which these effects of thimerosal occur requires further study.

Recent studies have suggested that survivin is positively regulated by Akt [66], [67]. Survivin has been implicated in the inhibition of apoptosis and is associated with a reduced apoptotic index in human tumor cells [68]. Similar to other mammalian inhibitors of proteins involved in apoptosis, survivin can bind to caspase-3 and caspase-7 [69]. Studies in a transgenic mouse model selectively expressing survivin in the skin confirmed that survivin inhibited the intrinsic, caspase-9-dependent apoptotic pathway [48], [70], [71]. Although some research has indicated that the expression of survivin is changed in accordance with the Akt pathway, the precise mechanisms by which survivin inhibits apoptosis in C2C12 cells have not been elucidated. Therefore, in this study we investigated whether the apoptosis-inducing activity of thimerosal is related to an alteration in survivin expression. Our results showed that down-regulation of survivin by thimerosal occurs in C2C12 cells and that it is accompanied by caspase-mediated apoptotic cell death. Furthermore, inhibiting the expression of survivin by siRNA induced cell apoptosis and sensitized thimerosal-induced apoptosis, and overexpression of survivin markedly suppressed thimerosal-induced apoptosis. As a pivotal component of multiple pathways, Akt has been shown to be a positive regulator of survivin expression in endothelial cells, and previous studies have claimed that the PI3K/Akt/survivin signaling pathway is an anti-apoptotic pathway in prostate cancer, lung cancer, myeloma, and leukemia [72], [73]. In our study in C2C12 myoblasts, we found that inhibition of Akt activation with wortmannin suppressed the expression of survivin and that activation of PI3K/Akt with mIGF-I increased survivin protein expression and could prevent the thimerosal-induced decreased expression of survivin. These results suggest that survivin is regulated by PI3K/Akt in C2C12 myoblast cells and that the PI3K/Akt/survivin signaling pathway plays an important role in thimerosal-induced apoptosis.

In conclusion, our work indicates that thimerosal-induced S phase cell cycle arrest and apoptosis of C2C12 myoblast cells occurs through the activation of mitochondrial signaling and that inhibition of the PI3K/Akt/survivin signaling pathway is involved in thimerosal-induced apoptosis in C2C12 cells. Although our work failed to clarify the mechanism by which thimerosal specifically regulates Akt, it suggests that thimerosal does not affect Akt directly. This unresolved question should be addressed in future research.
